# High Fidelity Simulations to Improve Pediatric Airway Management: A Narrative Review

**DOI:** 10.3390/healthcare14101357

**Published:** 2026-05-15

**Authors:** Alessandro Vittori, Cecilia Di Fabio, Marilena Trozzi, Corrado Cecchetti, Massimo Antonio Innamorato, Franco Marinangeli, Giuliano Marchetti, Marco Cascella

**Affiliations:** 1 Department of Anesthesia, Critical Care and Pain Medicine, ARCO, Ospedale Pediatrico Bambino Gesù IRCCS, Piazza S. Onofrio 4, 00165 Rome, Italy; 2 Department of Life, Health and Environmental Sciences (MeSVA), University of L’Aquila, Piazzale Salvatore Tommasi 1, Blocco 11, Coppito, 67010 L’Aquila, Italy; 3 Airway Surgery Unit, Bambino Gesù Children’s Hospital IRCCS, Piazza Sant’Onofrio, 4, 00165 Rome, Italy; 4 AUSL Romagna, Pain Unit, Department of Neuroscience, Santa Maria Delle Croci Hospital, 48121 Ravenna, Italy; 5 Surgery Unit, Bios Medical Center, Via Domenico Chelini 39, 00197 Rome, Italy; 6 Department of Medicine, Surgery and Dentistry, University of Salerno, Via Salvador Allende, 43, 84081 Baronissi, Italy

**Keywords:** simulation, high fidelity simulation, pediatric anesthesia, airway, airway management, laryngoscopy, cricothyrotomy, emergency, tracheostomy

## Abstract

High-fidelity simulations have become an important aid in clinical practice and research. In the pediatric field, they allow for the creation of scenarios involving emergency situations, in which decision-making algorithms must be rapidly applied, as is the case with airway management. Our narrative review examines articles in English indexed in PubMed, using the following search terms: high-fidelity simulation, pediatric, difficult airway management, bag mask ventilation, intubation, mechanical ventilation, tracheostomy, cricothyrotomy, laryngoscopy, fiberoptic bronchoscopy, and emergency situations. Airway management, along with venous access management, has always been one of the most challenging issues in pediatric anesthesia. The scarcity of patients and procedures, combined with the need to ensure high quality standards, necessitates simulations. Using high-performance devices and realistic settings allows us to reproduce not only the desired technical scenario, but also the emotions and group dynamics. High-fidelity simulations therefore prove to be an excellent aid for pediatric airway management, enhancing the hard and soft skills of both the team and the individual. Simulations allow us to replicate scenarios that are uncommon in clinical practice but have a significant impact due to their potential severity.

## 1. Introduction

In recent years, simulation has become fundamental for the development of both technical and non-technical skills in settings characterized by high clinical complexity, particularly when these settings are rare, unpredictable, or potentially catastrophic, thanks to the intrinsic features of high-fidelity simulation [1]. In the neonatal and pediatric setting, the use of high-fidelity mannequins capable of breathing, crying, and simulating seizure activity makes it possible to construct complex clinical scenarios that are consistent with situations involving a high cognitive and decisional workload. Pathophysiological parameters, cardiac and respiratory status, pulse oximeter readings, and cyanosis can be adjusted in real time via a remote laptop computer in response to learner actions and displayed on a standard neonatal monitor with appropriate alarms [2]. A relevant aspect has been highlighted in the field of neonatal resuscitation through the possibility of reproducing complex, infrequent, and emotionally demanding scenarios, which tend to place a greater burden on both the individual operator and the healthcare system. High-fidelity simulation is not intended solely as a tool for individual skills practice, but above all as a means to reveal operational criticalities within a team and to assess organizational readiness and the quality of the clinical response. It has been reported that pediatric residents are successful in less than 50% of intubation attempts [3,4,5], reinforcing the idea that simulation may bridge educational gaps by providing a context for improving effectiveness and safety. In pediatric patients, airway management represents a high-risk scenario in which even small anatomical or clinical variations may rapidly evolve into an emergency situation. High-fidelity simulation allows teams to prepare for extremely critical scenarios such as the “cannot intubate–cannot oxygenate” patient, an event that is rare in healthy children but, when it occurs, is often associated with poor outcomes [6,7]. The need to obtain emergency front-of-neck access in an infant or small child represents one of the most terrifying situations a clinician may face, and it is precisely in this context that high-fidelity simulation becomes a remarkable tool for training in life-saving procedures. In summary, this review is positioned within a framework in which simulation supports clinical training at multiple levels, from improving technical skills in airway management to strengthening non-technical skills, with the aim of reducing system-level vulnerabilities. The central theme of the review is that patient safety and team reliability may benefit from structured, repeated, and assessable simulation-based training, capable of transforming acquired competencies and rendering them “ready for use” in the event of emergencies. The primary objective of this review is to clarify the benefits of using high-fidelity simulations in pediatric airway management.

## 2. Materials and Methods

The present manuscript is a narrative review conducted through consultation of the PubMed database. This narrative review was conducted in accordance with the SANRA (Scale for the Assessment of Narrative Review Articles) recommendations to improve methodological transparency and scientific rigor [8].

The bibliographic search was carried out using a combination of keywords and MeSH terms, including high-fidelity simulation, pediatric, difficult airway management, face mask ventilation, intubation, mechanical ventilation, tracheostomy, cricothyrotomy, laryngoscopy, fiberoptic bronchoscopy, and emergency situations. Articles published in the English language on the management of difficult airways in the pediatric setting and on advanced procedures in critical and emergency contexts were included. After an initial selection based on title and abstract, the articles considered relevant were analyzed in full text and subjected to critical review. A supplementary search was performed by screening the reference lists of the selected papers to identify additional relevant studies. Further literature was identified based on the authors’ expertise.

Given the narrative purpose of the review and the heterogeneity of the available literature, the retrieved evidence was synthesized to provide a clinically oriented overview rather than a formal systematic appraisal.

We have structured our review with paragraphs following the order in which the airway is normally managed, even in emergency conditions.

## 3. Face Mask Ventilation

The management of the airway certainly constitutes a challenging moment in the care of pediatric and neonatal patients, especially in situations of respiratory emergency or during resuscitation maneuvers. The bag mask is the first device used in airway management and ventilation; its correct use is a fundamental competence that must involve all healthcare professionals engaged in the care of neonates and children. Despite the apparent simplicity of its use, mask ventilation is a complex procedure associated with numerous factors of failure and is strongly dependent on the operator’s experience; in clinical practice this skill is often poorly exercised, especially by non-specialist operators. For this reason, high-fidelity simulations become essential, as they allow the progressive acquisition, maintenance, and improvement of techniques related to pediatric airway management [9,10]. The anatomical characteristics of pediatric patients (relatively large occiput, excess soft tissue, and a larger tongue) make bag-mask ventilation particularly difficult [11]. In fact, despite adequate initial skills, 6.6% of otherwise healthy children are difficult to ventilate [12], increasing the risk of ineffective ventilation, considering that pediatric patients have a higher basal oxygen consumption and a lower pulmonary reserve compared with adults; all these elements are associated with a risk for pediatric patients of developing hypoxemia much more rapidly. Several studies have highlighted the difficulty faced by healthcare professionals in mask ventilation both in clinical practice and during simulations [13,14,15,16]. Among the main causes associated with ineffective ventilation are significant bag mask ventilation leaks, upper airway obstructions that result in the delivery of highly variable tidal volumes [17,18], and inadequate application of force to the face mask: excessive force may cause injury, airway obstruction, and operator fatigue, whereas insufficient force may result in leaks along the mask rim [18]. High-fidelity simulations represent a training method characterized by high fidelity of equipment, environment, and psychological aspects, which are necessary for training and learning in the management of pediatric and neonatal patients [19,20,21,22]. Simulation allows faithful reproduction of the clinical conditions in which mask ventilation occurs, thanks to mannequins designed to simulate variations in lung compliance, enabling operators to face difficulties that are as realistic as possible. Advanced simulators allow modulation of heart rate in relation to ventilatory performance and provide immediate feedback [23], thanks to the presence of video recordings. Simulations are particularly useful in mask ventilation, where the presence of small errors may be associated with clinical failure, not only to teach the technique but also to improve and refine the quality of ventilation by taking into account measurable and assessable parameters. Hannan et al. [24], in a randomized simulation study on the measurement of forces applied during positive pressure ventilation with a face mask, showed that overall expert operators applied a more uniform force along the rim of the mask compared with novices (median [Interquartile Range]: 0.46 [0.26–0.79] N vs. 0.65 [0.24–1.18] N, *p* < 0.001), and that such symmetry was associated with more effective ventilation. A limitation frequently observed during high-fidelity simulations in the management of mask ventilation was the lack of standardized mental models, particularly at the moment of initiating bag-mask ventilation in cases of impending respiratory failure. No care algorithm had been effectively implemented. This lack of standardization could be associated with suboptimal management of critically ill patients and could compromise clinical outcomes [25].

## 4. Laryngoscopy

Tracheal intubation, although considered a life-saving procedure in the emergency setting, is often associated with significant technical difficulties and a non-negligible risk of adverse events, especially in pediatric patients. In particular, neonatal intubation is one of the most complex procedures to perform and is characterized by low first-pass success rates and a high incidence of complications [26]. One of the main causes associated with the lack of consolidation of clinical and practical skills in the field of tracheal intubation that consistently emerges from the literature is the limited exposure, especially of residents and young physicians, to real pediatric intubations. Several studies [27,28] have shown that residents, in particular, have few opportunities to directly lead advanced airway management maneuvers even at the end of their training pathway [28]. A particularly relevant aspect for pediatric laryngoscopy in high-fidelity simulations is the possibility of simulating advanced pathological conditions such as tongue edema, laryngospasm, and trismus, up to the possibility of requiring the participant to perform a surgical airway [29]. All these characteristics typical of whole-body simulators make high-fidelity simulations ideal for training in stressful situations associated with a high risk of human error. In pediatrics, survival from an isolated respiratory arrest is greater than 75–80%, but in order to achieve this survival rate, efficient and appropriate airway management associated with a reduction in morbidity and mortality is necessary [30]. The difficulty of pediatric laryngoscopy is closely associated with the anatomical and physiological characteristics of pediatric patients. The main anatomical differences in infants and younger children are a prominent occiput, a relatively larger tongue compared with the pharyngeal space, floppy omega-shaped epiglottises, and more cranially located larynges; consequently, the pediatric population presents unique anatomical or functional difficulties in airway management [31]. From a physiological point of view, children have a limited pulmonary reserve associated with rapid desaturation during periods of apnea, making intubation time a determining factor for patient safety and the prevention of adverse events. Simulations make it possible to identify critical areas and important deficits in the management of pediatric airways, with the possibility of documenting harmful actions such as the administration of drugs for rapid sequence induction before preparing the intubation equipment, the bag-valve mask not connected to oxygen, an inappropriate endotracheal tube size, and the removal of a cuffed endotracheal tube with the cuff still inflated [29]. One of the most relevant contributions derives from a prospective observational study [29] (conducted on 16 pediatric residents in a medical simulation center) in which high-fidelity simulation was used to assess the skills acquired in airway management. In this study, 47 intubation attempts were performed with a number of successes equal to 27 (56%); it follows that even in a controlled simulated environment the success rate of pediatric laryngoscopy is just over half. The same simulation highlighted that appropriate preoxygenation, necessary to reduce the risk of significant desaturation during intubation, was performed in only 47% of cases (15 out of 32). Correct execution of the maneuvers involved in rapid sequence induction was documented in only 66% of cases (21 out of 32). With regard to the application of cricoid pressure, a maneuver frequently included in pediatric airway management protocols, it was applied in 63% of cases (20 out of 32). The use of an end-tidal carbon dioxide detector after placement of the endotracheal tube was observed only in a minority of simulations, in 34% of cases (11 out of 32). In the comparative, bicentric, randomized, and controlled study [32] conducted on a high-fidelity pediatric mannequin, normal airways and difficult airways managed either with the King Vision™ pediatric aBlade (KV) or with the C-MAC™ D-Blade were considered. The median [IQR] time to successful ventilation was significantly shorter for the KV. No differences were observed in first-pass intubation success rates (FPAs) between hyperangulated blades and direct laryngoscopes in the normal airway scenario, whereas in the difficult airway scenario, hyperangulated blades enabled FPAs ranging from 92% to 100% (KV), compared with 65% and 76% for conventional laryngoscopy (*p* < 0.001). A multicenter randomized study that included first- and second-year pediatric anesthesia residents demonstrated that simulations improved intubation skills regardless of fidelity levels [33]. The management of pediatric laryngoscopy in simulation was also evaluated in terms of perceived quality and educational impact within training programs, and the main result emerging from the study by Lejus-Bourdeau et al. [34] was the lack of a statistically significant difference in educational impact between high-fidelity pediatric simulators and low-cost simulators; obviously, the realism of the mannequin was considered greater in the high-fidelity group.

High-fidelity simulation can increase the success rate in laryngoscopy.

## 5. Intubation

The lack of time to achieve effective intubation without having physiopathological sequences constitutes one of the central risk factors in clinical practice, since despite the development of guidelines [35], difficult intubation remains one of the main causes of morbidity [36], especially in infants and young children due to their pathophysiological characteristics that predispose them to a more rapid onset of hypoxia during apnea [37]. During high-fidelity simulations, intubation is almost never proposed as an isolated technical management, but is always embedded within a potentially dynamic clinical scenario in which it is possible to modify the vital parameters of the simulated patient in association with decisions and maneuvers. The evolution of events such as dyspnea, apnea, obstruction, thoraco-abdominal asynchrony, laryngospasm, and bronchospasm appears on the monitor together with variations in hemodynamic parameters such as electrocardiogram and arterial pressure, and ventilatory parameters such as saturation, cyanosis, and respiratory rate [34]. The direct visualization of these variations associated with interruptions in ventilation or repeated intubation attempts reinforces the concept that time represents a critical variable in the management of pediatric patients. On the one hand, real opportunities to intubate a pediatric patient—and even more so a neonate—especially for physicians in training, have decreased [38,39,40]. On the other hand, the critical role of high-fidelity simulations in training has emerged, as they represent a compensatory tool, allowing the repetition of the procedure in a structured and standardized manner. Through simulations, residents especially achieved the required competencies. The acquisition of technical skills is associated not so much with technological realism as with exposure to the procedure itself. However, when simulations reproduce particularly stressful operating conditions, such as intubation during pediatric cardiopulmonary resuscitation, substantial differences emerge related to context and the devices used. In the study by Szarpak et al. [41], a simulation showed that intubation time was significantly longer with the traditional laryngoscope compared with the video laryngoscope, with intubation times (s) of 33 (24–36) versus 20 (17–23), and that the overall success of the procedure increased markedly (78.9% vs. 98.9%). In addition, both the potential trauma related to the procedure and the subjective difficulty of the procedure were significantly lower with the video laryngoscope. Obviously, it is not possible to determine the real impact that different devices may have on the quality of cardiopulmonary resuscitation or on patient outcomes, but first-attempt intubation success would certainly avoid the risk of trauma, edema, and bleeding associated with repeated intubation attempts [42,43]. Multiple intubation attempts are correlated with a high risk of failure, hypoxia, and even cardiac arrest [44]. Simulations can also be used as a tool to analyze in depth the interaction between operator and device during intubation, something that would be difficult and risky for patient safety in a real patient. This includes the quality of glottic visualization, ease of device use, and the cognitive load associated with the procedure itself [45]. High-fidelity simulations make it possible to review intubations through video recordings and to critically analyze the choices made, promoting deeper and more conscious learning that is not subject to individual biases associated with stress during situations of difficult management.

## 6. Fiberoscopy

The fiberoptic bronchoscope constitutes a cornerstone instrument in the advanced management of pediatric airways, considering the physiological and anatomical characteristics of the pediatric patient itself, which make tracheal intubation a high-risk procedure during which hypoxemia is more likely to occur compared with the adult patient [46]. Even brief delays in the management of the airway in children during general anesthesia can translate into severe critical events [47,48]. On the one hand, the videolaryngoscope can be considered a reliable instrument for the first attempt at intubation of a difficult airway [49]; on the other hand, visualization may be difficult because of limited mouth opening, mandibular hypoplasia, and syndromes associated with facial symmetry, or, even when visualization is optimal, it is not always easy to direct the tracheal tube toward the glottis due to insufficient angulation [50]. In this context, the fiberoptic bronchoscope is positioned as an essential instrument in cases of complex intubation, the use of which requires constant clinical training. The randomized and controlled study by Isogai et al., based on high-fidelity simulation, which compared the effectiveness of video-assisted fiberoptic intubation (VAFI) and traditional fiberoptic intubation in inexperienced operators, did not find statistically significant differences in intubation time or in the first-attempt success rate, with a mean of 55.0 s for fiber optic intubation (FOI) and 42.5 s for VAFI (*p* = 0.22). In the preliminary mannequin study [51], no statistically significant difference was detected in first-attempt intubation success between videolaryngoscopy (VL) and the fiberoptic bronchoscope, but significant differences were observed in the first-attempt success rate with VL, which were higher, mainly because attending anesthesiologists often do not have sufficient experience with advanced airway management devices.

Another useful technique in the management of difficult pediatric airways is the use of a SAD (a supraglottic airway device), which acts as a guide for fiberoptic intubation, with the advantage of allowing ventilation and oxygenation during the procedure [52,53], with a lower incidence rate of hypoxia, thereby maximizing safety in children and infants. Fundamental also in this case is the progression of the learning curve, which is maximal between the eighth and the twenty-fifth attempt, with a procedural success rate of 92.8% and a mean ± standard deviation procedural time of 71.3 ± 50.7 s [54].

In conclusions high-fidelity simulation can increase the success rate in fiberoscopy.

## 7. Emergency

Compared to adults, due to pathophysiological characteristics, children experience rapid hypoxia during periods of apnea [37], therefore a sequence of coordinated and technically correct actions is necessary. Despite the frequency in performing procedures, difficulty in intubation remains one of the major causes of mortality [36,55]. Intubation is not always a planned procedure under controlled conditions, especially in the emergency setting, where it often constitutes the response to progressive deterioration. Conditions such as severe bronchiolitis, ingestion of sedatives, extensive burns, or neurological impairment are often associated with difficulty in ventilation through non-invasive methods, requiring advanced support. In high-fidelity simulation settings these deteriorations can be dynamically reproduced, allowing the evaluation of operators’ responses. In the prospective observational study by Frank L et al. [29] two scenarios to be managed were considered: the first, a 3-month-old child with severe bronchiolitis and respiratory distress evolving toward respiratory failure; the second, a 16-year-old adolescent with alcohol intoxication and respiratory depression. It was reported that adequate preoxygenation had been provided only in 47% of cases; this value is truly critical since in more than half of the cases adequate preoxygenation was not performed, compromising the respiratory reserve and drastically reducing tolerance to pharmacologically induced apnea. Emergency airway management instead requires that the decision to intubate be accompanied by complete anticipatory preparation with monitoring, ready suction, dosed medications, and verified equipment, including accuracy in the selection of the endotracheal tube (ETT), cuffed or uncuffed, its positioning, depth, and choice. The prospective study by Sultaneh et al. [56] highlighted that the execution of high-fidelity simulations significantly improved decision-making choices, including appropriateness in the selection of the endotracheal tube (ETT) (from 67% to 100%, *p* = 0.02) and of the cuffed endotracheal tube (from 8% to 71%, *p* < 0.001). These data indicate that initially approximately one third of participants selected an inappropriate ETT, a choice that especially in the emergency setting could be associated with ineffective ventilation, leaks, increased resistance, or laryngeal trauma. Achieving intubation on the first attempt is a crucial objective in pediatric airway management since each additional attempt increases the risk of desaturation, aspiration, and hemodynamic instability. Again, in the study by Overly et al. [29] the analyzed data reported 47 attempts at intubation with 27 successes (56%). A success rate of 56% implies that almost half of attempts fail, which from a clinical point of view is extremely relevant because in a critically ill child even a single failed attempt can result in a significant drop in oxygen saturation. In the same study, evaluation of correct tube placement was also not systematic, but the use of the end-tidal CO_2_ detector occurred in only 34% of cases. Failure to use the exhaled CO_2_ detector compromises procedural safety because prolonged unrecognized esophageal intubation in a pediatric patient may be associated with cardiovascular collapse if not promptly identified. The management of pediatric emergencies becomes more complex in centers with a low volume of pediatric cases; in the United States 34 million pediatric visits to emergency departments occur each year, across 5000 emergency departments, of which 4 million involve high-acuity patients requiring emergent and life-saving interventions [57]. Significantly more adverse events occur in pediatric airway management in community hospitals compared to tertiary pediatric emergency departments [58]. Reduced clinical exposure translates into decreased familiarity with pediatric emergencies and a greater likelihood of procedural errors. In emergency pediatric airway management, error is almost never associated with a single isolated act but is often caused by clinical, organizational, or human factors: the majority of errors occur due to breakdowns in communication or teamwork [59,60,61]. Ineffective communication may result in errors in equipment preparation, in the pharmacological sequence, or in post-intubation management, especially in the intensive care setting where the high acuity of illness and the complexity of medical care increase the risk of errors [1]. In this context, the essential role is that of the team leader whose task is to minimize risk. High-fidelity medical simulations are an effective tool to develop team leader competencies [62,63].

## 8. Tracheostomy and Cricothyrotomy

Pediatric tracheostomy represents a complex and high-risk clinical condition associated with high morbidity and mortality, with complication rates ranging between 12.6% and 30% in children and a 5-year tracheostomy-associated mortality ranging between 1% and 8% [64,65,66]. Tracheostomy-related complications, including accidental decannulation or tube obstruction, may rapidly evolve into critical scenarios requiring prompt assistance with significant teamwork in order to prevent hypoxic brain injury and mortality [67,68]. One of the recurring elements in the literature is the presence of significant educational gaps among non-otolaryngologist healthcare professionals involved in the management of these patients, who present knowledge deficits and discomfort in tracheostomy management [69,70]. However, knowledge and understanding of tracheostomy management should also be expanded among healthcare professionals who routinely care for patients with tracheostomies [71,72,73,74]. These critical issues are particularly relevant in the intra-hospital setting, where the first response to an emergency is not always guaranteed by dedicated specialists, and pediatric residents often find themselves acting as first responders in the event of a tracheostomy emergency [75]. Clinical simulation emerges as an important educational tool aimed at improving multidisciplinary team dynamics and the management of critical events, allowing healthcare professionals to operate within their real clinical environments using tools and resources that are routinely available [76,77]. In the context of resident training, the described programs include didactic modules integrated with practical sessions that lead to improved knowledge in the management of tracheostomy and related emergencies. Objective knowledge test results significantly increased, from a pre-test mean percentage of 61.25 (SD = 13.52) to a post-test mean percentage of 81.25 (SD = 10.57) [t(39) = −8.144, *p* < 0.001] [75]. In the context of extreme airway management emergencies, pediatric cricothyrotomy and emergency front of neck access represent rare procedures; therefore, providers tend to lose proficiency in performing the procedure itself [78,79]. Despite the need for training, the literature provides discordant guidance on how to train for and perform emergency front of neck access as a life-saving measure in children younger than 8 years of age [71,80,81]. Cannula insertion in a pediatric “cannot intubate–cannot oxygenate” situation is questionable due to the high failure and complication rates [82,83,84]. The study by Ulmer et al. [85] analyzed the learning curves of 50 physicians from five different specialties who performed 10 emergency tracheotomies on rabbit cadavers after watching an instructional video. With an overall success rate of 94%, performance time decreased from 107 s (standard deviation [SD], 45) to 55 s (SD 17) over 10 attempts. The learning curve was steep between the first and fourth attempt, with an 11% reduction in performance time. In the clinical literature, structured high-fidelity simulation programs specifically dedicated to the integrated management of pediatric tracheostomy and cricothyrotomy are not described; different types of simulation have been considered, including the use of pediatric mannequins, animal models, and low-cost models.

## 9. Discussion

Simulations are increasingly becoming an indispensable tool in clinical practice. They allow for the replication of difficult or emergency scenarios, allowing clinicians to train in unfamiliar contexts [86]. In pediatrics, this concept is particularly important, as pediatric procedures represent a small portion of surgical and anesthesiologic procedures. Given the declining birth rate we are seeing in Western countries, this takes on even more importance because every year the number of pediatric procedures is drastically reduced, especially in small centers. High-fidelity simulations can reproduce emergency situations as close to reality as possible and can provide ongoing training for the teams involved. However, simulations have limitations and are not a panacea. The first and most significant limitation is cost. Building a high-fidelity simulation center requires a significant initial investment, not only in terms of space and equipment, but also in initial staff training. This makes it difficult to use high-fidelity simulations in developing countries. Another major issue concerns the training of simulation staff. It is often said that simulation is an excuse for debriefing, and that this is therefore the true core of simulation. Only trained staff capable of managing debriefing intelligently and constructively can transform simulation into an opportunity for learning and team building.

## 10. Limitations

The main limitation of our manuscript is its narrative review nature. However, this limitation can be transformed into a strength, given that a narrative review is more accessible than a systematic review for those approaching this topic for the first time. Another major limitation is that the scientific literature focused strictly on pediatrics is always less robust than that based on adult populations. This is a limitation for any pediatric topic. However, by drawing on the existing pediatric literature, it is possible to apply, with the right critical eye, evidence that is not strictly pediatric.

## 11. Conclusions

High-fidelity simulations are now becoming routine clinical practice, especially in areas with limited caseloads. This is precisely the case for pediatric anesthesia, where the number of procedures is gradually declining due to the declining birth rate in some regions. The other major advantage of high-fidelity simulations is the ability to reproduce truly realistic situations and therefore replicate the psychological impact. In pediatric anesthesia, the psychological impact, except in emergency situations, constitutes a significant challenge for the physician. High-fidelity simulations are therefore a valuable tool for training the physician and the team, both in technical skills and soft skills. Additionally, high-fidelity simulations are an important tool for team building.

## Data Availability

No new data were created or analyzed in this study. Data sharing is not applicable to this article.
